# Retinal arterial macroaneurysm rupture by Valsalva maneuver: a case report and literature review

**DOI:** 10.1186/s12886-022-02662-x

**Published:** 2022-11-30

**Authors:** Yang Meng, Yishuang Xu, Lu Li, Lu He, Zuohuizi Yi, Changzheng Chen

**Affiliations:** 1 Department of Ophthalmology, Renmin Hospital of Wuhan University, 238 Jiefang Road, Wuhan, China; 2 Physical Examination Center, Renmin Hospital of Wuhan University, 238 Jiefang Road, Wuhan, China

**Keywords:** Retinal arterial macroaneurysm, Valsalva maneuver, Retinal diseases, Case report, Review

## Abstract

**Background:**

Retinal artery macroaneurysms (RAMs) are focal dilations of the large retinal arteries. Most RAMs are asymptomatic, however, when hemorrhage or exudation caused by a RAM involves the macula, patients can experience marked vision loss. This article reported a rare case of a ruptured RAM due to the Valsalva maneuver in an elderly female with constipation and offered a review of the relevant literature.

**Case presentation:**

A 78-year-old woman with several risk factors presented with multi-level retinal hemorrhages following a Valsalva maneuver during constipation. Due to the poor coagulation and heavy bleeding in this case, the blood broke through the internal limiting membrane and drained "on its own" into the vitreous cavity. First, we observed the patient and controlled for her risk factors. After the blood was completely drained into the vitreous cavity, the root cause of the bleeding was found to be a RAM rupture. After laser photocoagulation, the patient's vision recovered significantly and remained stable for a long time despite the presence of an epiretinal membrane and a lamellar macular hole.

**Conclusions:**

This is the first reported case of a RAM rupture by Valsalva maneuver during constipation. For multi-level hemorrhages caused by RAM, measures should be taken to drain out the sub-internal limiting membrane hemorrhage and simultaneously control for risk factors. After the RAM is exposed, laser photocoagulation can be performed.

## Background

Retinal arterial macroaneurysms (RAMs) are acquired focal dilatations of the large retinal arteries, usually occurring within the first three bifurcations of the central retinal artery [1]. Typically, RAMs are seen in elderly women and are usually closely related to long-standing hypertension and arteriosclerosis [2]. Most RAMs are located in the temporal retina, and many may regress spontaneously with a favorable prognosis [3, 4]. However, a marked decrease in visual acuity may result when the macula is involved by hemorrhages and exudates. The Valsalva maneuver is a forced expiration against a closed glottis causing transient systemic changes such as increased blood pressure and intraabdominal pressure [5]. It is quite common in daily life and typical activities such as coughing hard, vomiting, straining, constipation, and weightlifting can all lead to it [6]. The Valsalva maneuver can affect retinal arteries due to the sudden and violent fluctuation of blood pressure that occurs [7].

To the best of our knowledge, there have yet to be any reports of RAM rupture by a Valsalva maneuver-related mechanism. Here, we reported a rare case of the rupture of a retinal arterial macroaneurysm by the Valsalva maneuver in an elderly female with hypertension and constipation and provided a review of the relevant literature.

## Case presentation

**Fig. 1:**
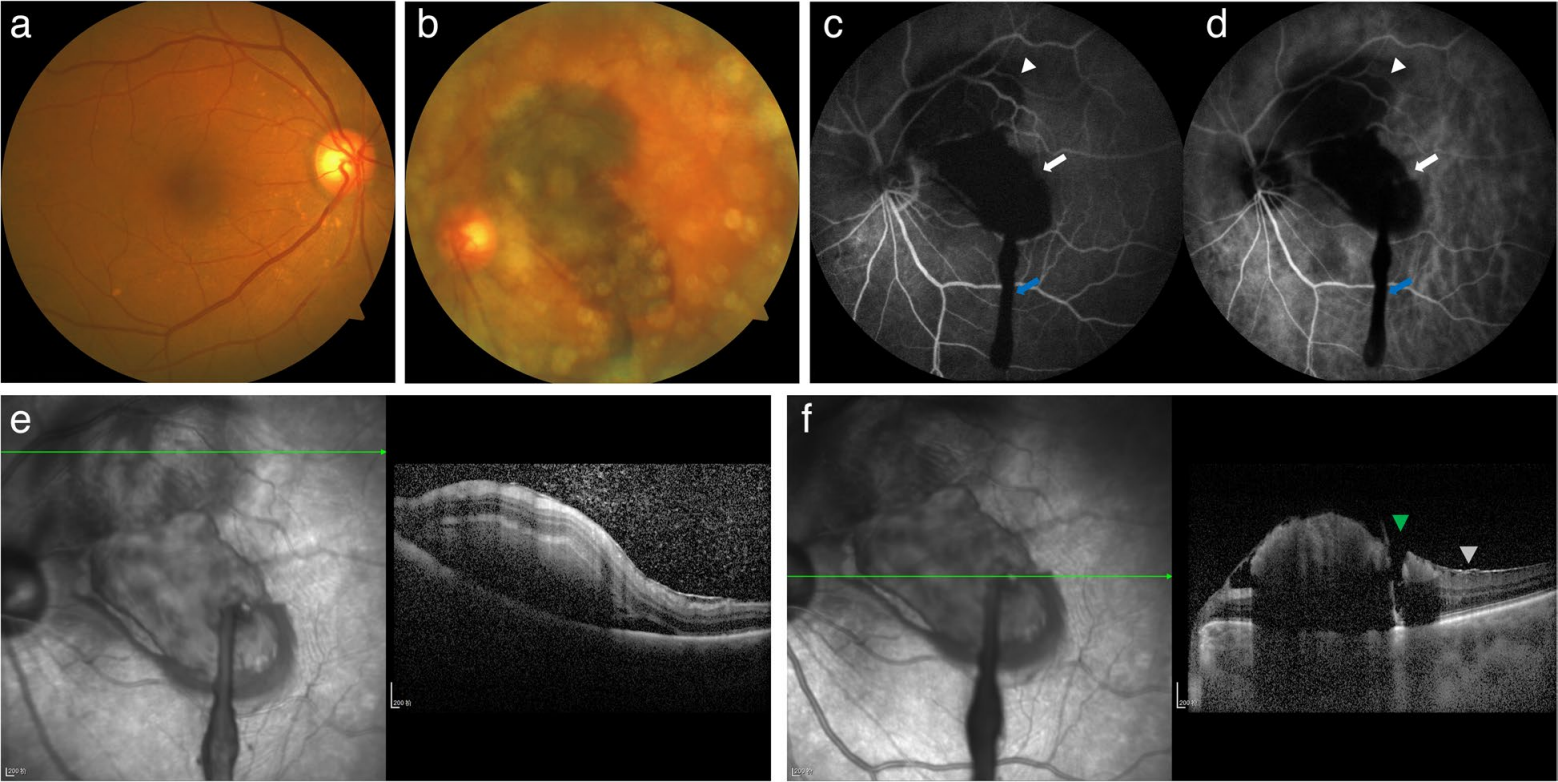
CFP, FFA, ICGA, and SD-OCT examinations of the patient on the first visit. CFP showed drusens scattered at the posterior pole of the right eye (**a**) as well as retinal hemorrhage, vitreous hemorrhage, and asteroid hyalosis in the left eye (**b**). In FFA (**c**) and ICGA (**d**), subretinal hemorrhage in the upper retina (arrowhead), sub-ILM hemorrhage in the macular area (white arrow), and strand-shaped VH (blue arrow) could be distinguished. SD-OCT with infrared image showed a fusiform lesion with a hyporeflective area under a hyperreflective band between the neurosensory retina and retinal pigment epithelium, according with subretinal hemorrhage (**e**). SD-OCT and infrared images demonstrated the presence of ERM (grey arrowhead), an arched hemorrhage below the ILM, and a rupture of the ILM (green arrowhead) in the macular area (**f**)

A 78-year-old female was admitted to our department for sudden-onset painless vision loss, central scotoma, and an “earthworm-shaped” floater in her left eye. These symptoms were noticed after she had a hard bowel movement the prior night. Examination demonstrated the best-corrected visual acuity (BCVA) of 20/40 in her right eye and finger counting in her left eye. Intraocular pressures were 16 mmHg and 14 mmHg in her left and right eye, respectively. A history of hypertension for 15 years was reported and she had been on oral antihypertensive medication. Her blood pressure at presentation was 146 over 83 mmHg. She had persistent atrial fibrillation for 5 years, so she was on long-term oral rivaroxaban (10 mg QD; Xarelto, Bayer Pharma AG). Moreover, 2 years ago, the patient had two coronary stents implanted due to coronary heart disease and she was taking oral aspirin (100 mg QD; Bayaspirin, Bayer Pharma AG). The patient also had a history of untreated constipation for more than 10 years. Any history of ocular disease or trauma was denied. Her eye movements, pupillary responses, and anterior segment examination were unremarkable in both eyes. Dilated fundus examination of the right eye showed drusens scattered at the posterior pole and examination of the left fundus showed the presence of asteroid hyalosis, vitreous hemorrhage (VH), as well as subretinal and sub-inner limiting membrane (sub-ILM) hemorrhage. Color fundus photographs (CFP) revealed that the strand-shaped appearance of VH was consistent with the patient's chief complaint of an “earthworm-shaped” floater (Fig. 1a and b). Fundus fluorescein angiography (FFA) and indocyanine green angiography (ICGA) showed the presence of retinal blood vessels in the upper part of the hemorrhage while those in the lower part were covered, representing subretinal and sub-ILM hemorrhage, respectively (Fig. 1 c and d). These two retinal hemorrhages were also clearly identified in Spectral-domain optical coherence tomography (SD-OCT; Spectralis HRA + OCT; Heidelberg Engineering, Heidelberg, Germany) (Fig. 1e and f). In addition, the epiretinal membrane (ERM) and a rupture of the inner limiting membrane (ILM) were noted at the junction of the VH and the sub-ILM hemorrhage in the macula (Fig. 1f). Blood chemistry tests show elevated triglycerides (2.1 mmol/L). Her blood cell count was normal, but the prothrombin time (PT) and activated partial thrombin time (APTT) were delayed (30.1 s and 45.8 s, respectively). The international normalized ratio (INR) was elevated (2.82). Based on her clinical presentation and history, a preliminary diagnosis of epiretinal membrane and Valsalva maneuver-related retinal hemorrhage and VH was made.

**Fig. 2:**
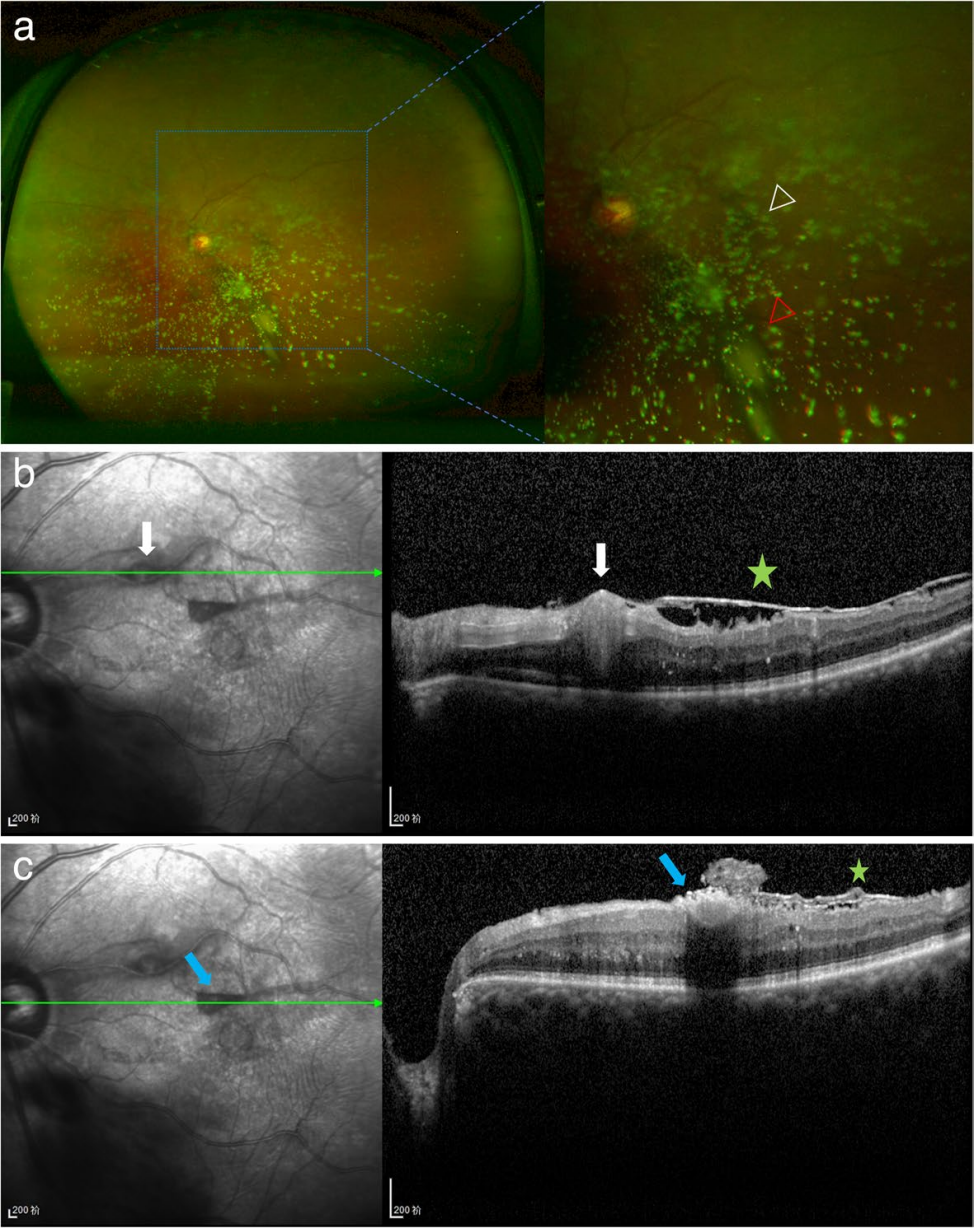
Examinations of the patient 6 weeks after initial vision loss. The fundus photograph taken with the Optomap® camera and an amplified view of the fundus photograph showed a small fraction of VH (red arrowhead) and retinal hemorrhage (white arrowhead) that had not been absorbed (**a**). The RAM (white arrow) was discernible on the B-scan and IR images (**b**). B-scan and IR images showed the residual sub-ILM hemorrhage (blue arrow) covering the retina underneath (**c**). Both B-scans showed continuous band-shaped hyperreflective signals (pentagram) on the retinal surface, consistent with ERM (**b** and **c**)

Because part of the retinal hemorrhage had drained into the vitreous cavity through the rupture of ILM, and considering the advanced age and concomitant diseases of the patient, regular follow-up examinations on ocular condition were advised. A careful dilated fundus examination was performed at each visit to observe the absorption of the hemorrhage and to facilitate the timely intervention for any adverse progression (All follow-up examinations were performed by Y.M., L.L., and L.H.). The patient was simultaneously referred to the department of cardiology for regulatory medication to control blood pressure, triglycerides, and the risk of bleeding. After six weeks, most of the retinal hemorrhage had been absorbed, with the exception of a small amount of the sub-ILM hemorrhage which was left in the supranasal macula (Fig. 2a). The infrared (IR) and OCT B-scan images revealed a retinal arterial macroaneurysm on the temporal side of the optic disc at the posterior pole, with a large area of ERM covering the posterior pole and minor sub-ILM hemorrhage (Fig. 2b and c). The BCVA was 20/40 in her right eye and 20/80 in her left eye. The patient and her family required quick and safe recovery of vision. After comparing the potential risks, benefits, and costs of surgery with laser and other treatments, they chose laser treatment. To speed up the regression of the RAM, 577-nm yellow laser photocoagulation (NIDEK MC-500 Vixi, Nidek, Gamagori, Japan) with 180 mW power was carefully performed at the RAM (by Y.X.). The duration was 0.2 s and the spot size was 200 μm using a single pulse. The patients did not report any discomfort after laser treatment.

**Fig. 3:**
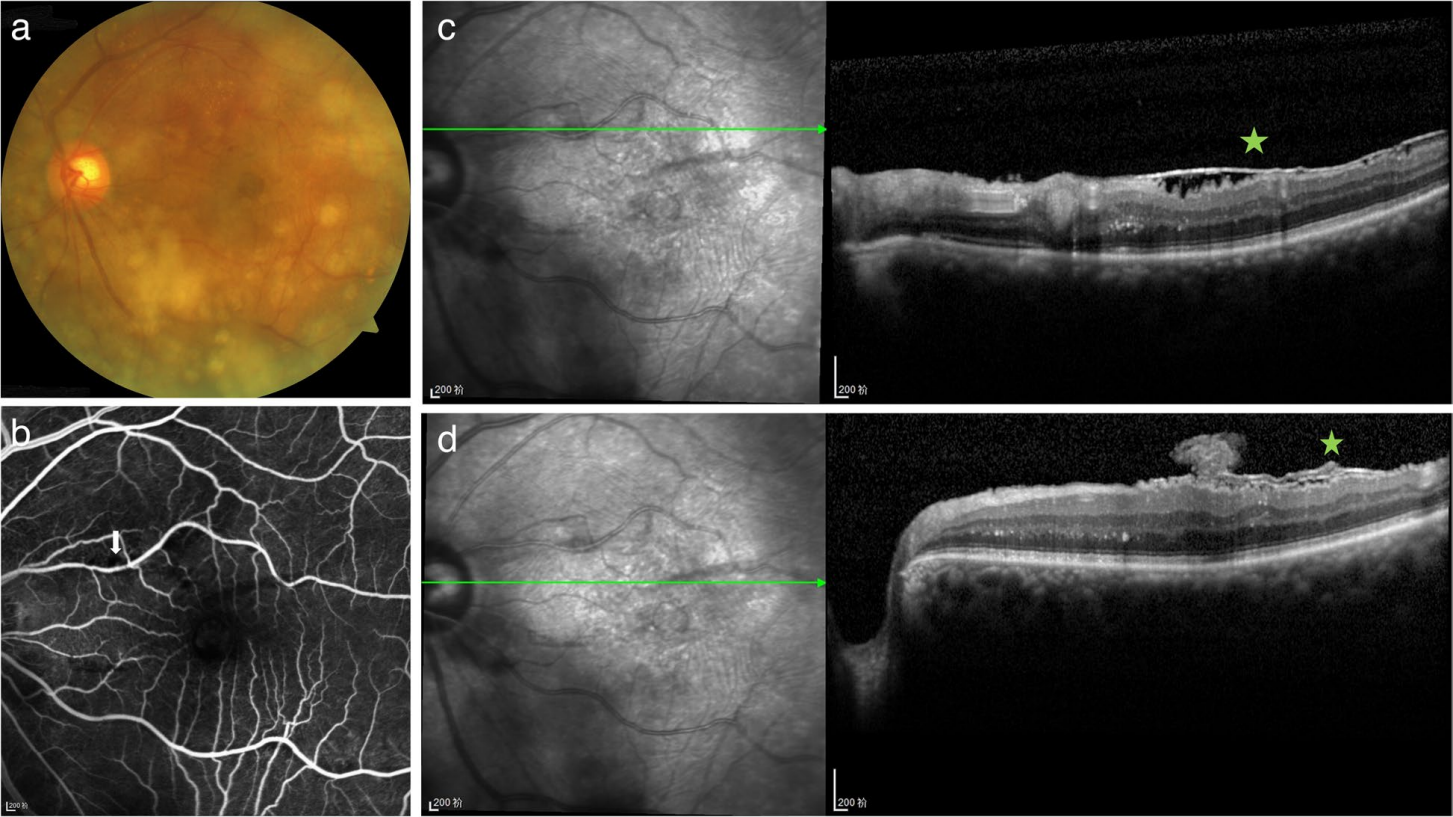
Examinations of the patient 9 weeks after first visit (3 weeks after laser photocoagulation). CFP indicated that almost all of the hemorrhages at the posterior pole had been absorbed (**a**). After laser photocoagulation, the RAM (white arrow) was closed on FFA (**b**). SD-OCT demonstrated RAM shrinkage (**c**) and complete absorption of the residual sub-ILM hemorrhage (**d**). The ERM (pentagram) was stable (**c** and **d**)

**Fig. 4:**
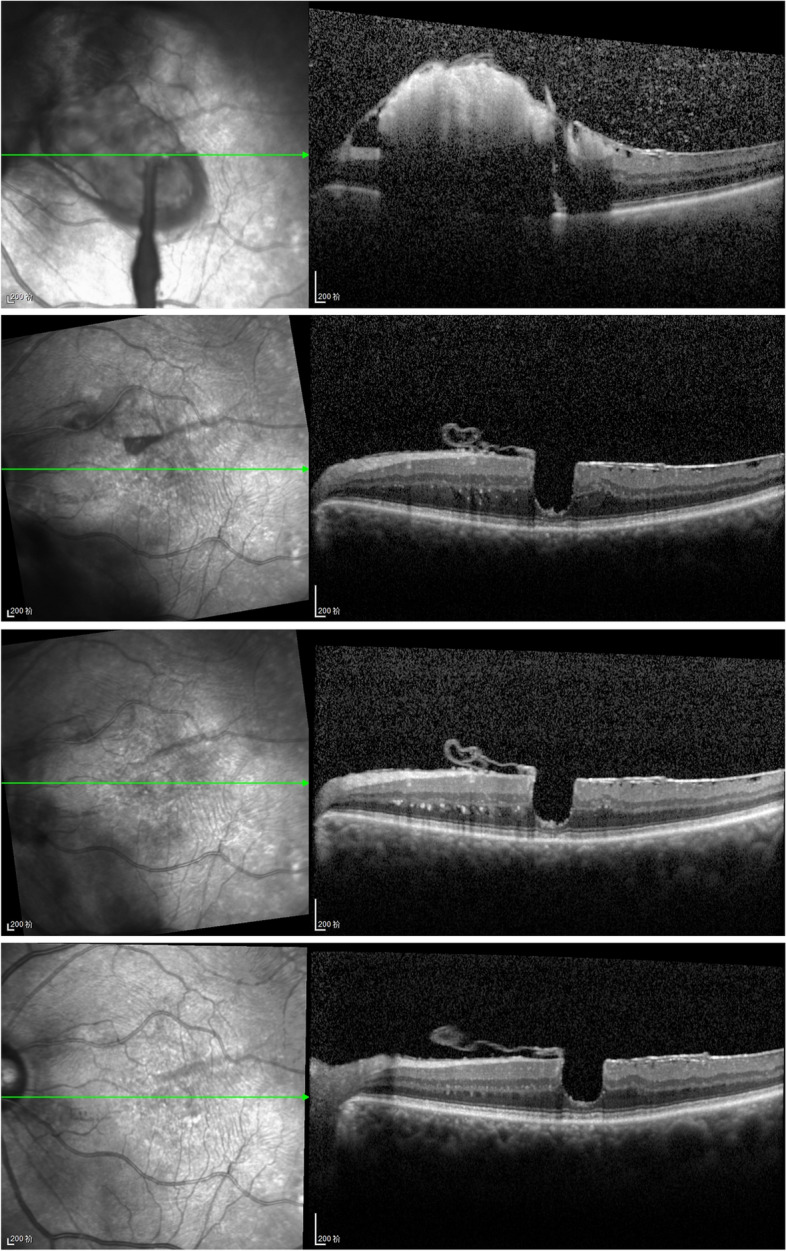
IR and B-scan images of the same position from the patient's first visit to 10 months later. The ERM and lamellar macular hole had remained stable since the 6th week

Nine weeks after her initial visit, all retinal hemorrhages had been absorbed (Fig. 3a), and the patient's BCVA also improved to 20/40 for both eyes. CFP and SD-OCT showed a significant regression of the RAM following the laser photocoagulation (Fig. 3a and c). FFA confirmed the closure of the RAM without affecting the surrounding retinal vessels (Fig. 3b). Although ERM had been found since her first visit, it was observed without recommendation for treatment because it was not the main problem threatening the patient's vision at the current time. Six weeks after her initial visit, the formation of a lamellar macular hole was found just at the junction of the previous VH and sub-ILM hemorrhage. The ERM and lamellar macular hole remained stable during her subsequent follow-up visits (Fig. 4). Thus, the main diagnosis of this patient was RAM rupture induced by the Valsalva maneuver. Her ophthalmological manifestations remained stable for 8 months after the final diagnosis (Fig. 4). No adverse or unanticipated events had been observed till her latest follow-up. The patient was satisfied with the treatment effect.

## Discussion

### Epidemiology

There is no authoritative data yet on the incidence rate of RAMs, but they are most frequently seen in people over 60 [8, 9]. According to published literature, women account for 70% ~ 78% of RAM cases [8, 10–12]. The mostly reported location of RAMs is the superotemporal quadrant of the retina, partly because RAMs at this location are more likely to show visual symptoms [3, 13].

### Risk factors

Known risk factors of RAMs include female sex, advanced age, systemic hypertension, hypercholesterolemia, polycythemia, and arteriosclerotic cardiovascular disease, among others [1, 9, 14–16]. Previous studies have suggested that hypertension is the most important risk factor, and the proportion of hypertension in patients with RAMs ranges from 51 to 75% [11, 12, 17]. Although there is no ocular risk factor that has been clearly confirmed by previous studies, several ocular diseases have been reported to be associated with RAMs, including retinal venous occlusion (RVO), retinal arterial occlusion (RAO), and macular hole (MH) [14, 18–20].

### Pathophysiology

The precise pathophysiology of RAMs has not been well-understood to date. However, it has been recognized that RAMs are characterized by thinned blood vessel walls, decreased elasticity, and fibrosis secondary to the loss of the muscular coat, thus leading to increased susceptibility to dilatation by raised intraluminal pressure [14, 15]. The focal weakness of the arterial walls results in RAMs. And rupture of RAMs occurs when intravascular pressure exceeds the threshold that the fragile vessel walls can withstand. From this perspective, various focal or systemic factors leading to increased vessel wall susceptibility may be responsible for RAMs. For example, hyaline degeneration and arteriosclerosis of the retinal arteries are commonly seen in patients with sustained hypertension, and these conditions can partly explain why hypertension is the most important risk factor for RAMs [17, 21].

### Clinical manifestations

The presentation of a patient with RAMs can be variable. RAMs can be classified into three types: hemorrhagic, exudative, and quiescent, between which the management and prognosis may differ [3]. Hemorrhagic RAMs are caused by the acute rupture of the fragile retinal artery walls. Depending on the location of the rupture, hemorrhagic RAMs can cause multi-level retinal hemorrhages and can be observed in the preretinal, sub-ILM, intraretinal, and subretinal spaces [21, 22]. VHs occur when blood breaks through the ILM and is drained into the vitreous. Exudative RAMs are marked by exudates with or without macular edema. If a RAM shows both exudates and hemorrhages, the type is based on the initial predominant complication causing decreased visual ability (VA) [2]. Contrary to the first two types, quiescent RAMs are generally asymptomatic with no obvious hemorrhages or exudates. However, hemorrhages and/or exudates could also be found in quiescent RAMs when the macula is not affected [2, 16]. Quiescent RAMs may develop into the hemorrhagic or exudative type. Typically, RAMs are unilateral, but there are reports of bilateral RAMs or multiple RAMs occurring in the same eye [23, 24]. Neurosensory retinal detachment and macular edema with or without exudations can also be seen in some cases [4]. RAMs might be found upon routine ophthalmic examination in patients without any ocular symptoms. However, most RAMs are found because of the abrupt and severe decrease in VA and loss of visual field that result from hemorrhages and exudates [11, 25, 26].

### Diagnosis and differential diagnosis

The diagnosis of RAMs is typically made based on clinical manifestations, demographic characteristics, and examinations such as FFA, ICGA, and OCT [16, 26–28].

However, RAMs can be described as a masquerade syndrome and may mimic a range of other ocular diseases [29]. According to previous literature, RAMs are frequently misdiagnosed, especially when the underlying RAMs are obscured by hemorrhages or exudates [3, 14]. Considering that these studies were completed at a time when RAMs were not well understood and the means of inspection were more limited and less advanced, the current misdiagnosis rate should now be lower. Yet, it is still necessary and important to distinguish RAMs from other mimickers in clinical practice. For patients with retinal hemorrhages, mimickers such as RVO, diabetic retinopathy (DR), retinal telangiectasia (Coats’ disease and Leber's miliary aneurysms), Valsalva retinopathy, idiopathic polypoidal choroidal vasculopathy (IPCV), choroidal neovascularization (CNV), retinal capillary hemangiomas, and retinal cavernous hemangioma need to be excluded [15, 30, 31]. In patients with lesions covered with dense VH, mimickers including wet age-related macular degeneration (wAMD), proliferative diabetic retinopathy (PDR), retinal tear, and ischemic RVO should be ruled out before making a diagnosis [15, 32].

### Treatment

A wide range of treatment options for RAMs is available and has been reported in previous literature, including observation, laser photocoagulation, Nd: YAG laser membranotomy, anti-vascular endothelial growth factor (anti-VEGF) therapy, surgery, and tissue plasminogen activator (tPA) [32–36]. However, there is not yet a widely recognized treatment protocol. Moreover, treatment options should be tailored to the patients' specific conditions. Despite this, controllable risk factors including systemic hypertension, hypercholesterolemia, polycythemia, and arteriosclerotic cardiovascular disease must be controlled regardless of which management option is chosen.

#### Observation

It should be noted that a large part of RAMs are of no severe threat to vision and may regress spontaneously with a favorable prognosis [14, 32]. For asymptomatic patients without hemorrhages or exudations related to RAMs, a visit every six months until regression would suffice [4]. It is suggested that patients without macular involvement should be observed, and those with vision loss secondary to vitreous, intraretinal, or preretinal hemorrhages (macula not involved) may also be observed for spontaneous regression for a few months before the treatment [32, 37].

#### Laser photocoagulation

Laser photocoagulation has been used in the treatment of RAMs since the 1970s and has obtained satisfactory results [33, 38]. Both direct and indirect laser photocoagulation treatment can be used, especially when exudation or edema is vision-threatening. Direct laser photocoagulation is performed directly at the RAM, aiming to speed the regression of the RAM and fundamentally reduce the leakage [4]. As another option, indirect laser photocoagulation is applied to the surrounding retina of the RMA to order to keep the leakage away from the macula [33]. The main complications of laser photocoagulation are arteriolar occlusion, retinal traction, increased exudation, capillary dropout, and subretinal scarring [4, 39]. In recent years, subthreshold laser treatment (STLT) has been proposed to reduce the side effects of conventional laser photocoagulation [40, 41]. By reducing the duration of laser exposure and using sub-visible clinical endpoints, STLT can achieve similar treatment outcomes while reducing the complication rate when compared with conventional laser photocoagulation [40, 41].

#### Nd: YAG laser membranotomy

For sub-ILM hemorrhage secondary to RAM rupture, Nd: YAG laser membranotomy should be considered as a method for draining the sub-ILM hemorrhages into the vitreous for quicker absorption and has been proven to be both safe and effective [22]. It has been suggested that, once it is considered, Nd: YAG laser should be performed as early as possible because complete drainage is hard to achieve if the blood has become clotted [42]. Major complications of Nd: YAG laser include non-clearing VH, retinal detachment, macular hole, and ERM formation [43, 44].

#### Anti-VEGF therapy

Intravitreal anti-VEGF therapy for RAM treatment was first introduced in 2009 [36]. Since then, anti-VEGF therapy has been frequently studied as a promising treatment for RAMs with hemorrhage or macular edema. Anti-VEGF agents may close the involved pathologically permeabilized retinal arteries by normalizing the vessel walls and inducing thrombosis within the RAMs [11, 45]. Several previous studies indicate that anti-VEGF agents are an effective therapy for complicated hemorrhagic and/or exudative RAMs, resulting in quick and safe improvement of vision with fewer complications [11, 45, 46]. It has also been noted that combined laser and anti-VEGF therapy can rapidly reduce macular exudation by RAM [22]. However, the spontaneous nature of RAM regression may affect the reliability of the conclusions made in previous studies. A recently published study with a relatively large sample size compared the long-term treatment outcomes of hemorrhagic RAMs treated with anti-VEGF therapy or observation alone [12]. The findings were that there was no significant difference in the BCVA between the observation group and the anti-VEGF group [12]. Further studies are needed to fully reveal the influence of anti-VEGF therapy on VA. Although anti-VEGF agents were proven to be safe, the complications of intravitreal injections should be noted, including but not limited to endophthalmitis, VH, subconjunctival hemorrhage, retinal detachment, and retinal tear [47].

#### Pars plana vitrectomy

Although laser photocoagulation is a widely chosen treatment choice for RAMs, pars plana vitrectomy (PPV) should be considered in the following cases: (1) submacular hemorrhages that cannot be treated by laser photocoagulation; (2) non-clearing VHs after observation for over three months, as the natural resorption process usually lasts less than three months; (3) VHs that obscure the macula, making the diagnosis difficult, especially when other lesions are suspected; (4) VHs that interfere with necessary treatment needed for vision recovery; and (5) removal of pre-retinal hemorrhages if quicker vision recovery is needed [4, 32, 35, 37, 48]. In appropriate circumstances, PPV can help to remove the pre-retinal hemorrhages in a timely manner, reducing the toxic effects on the retina and reducing the probability of ERM formation [43, 48, 49]. Still, a substantial number of reported cases achieve satisfactory results with observation or laser, and the pros and cons must be fully weighed before performing PPV [30, 38, 50]. Complications associated with PPV include retinal tear, progression of cataracts, retinal detachment, and recurrent VH [35, 51].

#### tPA combined submacular surgery/pneumatic replacement

The combination of tPA with submacular surgery or pneumatic replacement has been effective in the treatment of submacular hemorrhage (SMH) [52–54]. Submacular surgery includes surgical drainage of the submacular hemorrhage through retinotomy, fluid-gas exchange, and insufflation with intravitreal gas [4, 53]. Pneumatic displacement is less invasive and includes perfluorocarbon gas injection and downward gaze positioning in order to remove the hemorrhage from the macula [54]. These two approaches both require patients to adopt a face-down position for several days after the operation. The prompt displacement of the SMH can reduce the macular damage caused by the hemorrhage and contribute to the effective recovery of VA [52]. Potential complications of pneumatic replacement include failure of displacement, VH, endophthalmitis, etc. Complications of submacular surgery are similar to those of PPV.

### Case summary

This case is special, as the patient with RAM rupture suffered from multi-level hemorrhages likely due to a Valsalva effect related to constipation. She was under antiplatelet therapy and had nearly all of the aforementioned risk factors: female gender, old age, long-standing systemic hypertension, hypercholesterolemia, and arteriosclerotic cardiovascular disease. The combined effect of these risk factors had increased the susceptibility of her arterial walls. Usually, the blood pressure is supposed to be the lowest at night, when the patient was straining on the toilet, this caused her to experience greater blood pressure fluctuations during the Valsalva maneuver [55]. The rupture of the RAM occurred when the huge fluctuations in blood pressure hit her already fragile vessel walls. In this case, we initially opted for regular observation on her ocular condition because the blood had broken the ILM and flowed into the vitreous, equaling to an Nd: YAG laser membranotomy. At the same time, the relevant risk factors had also been strictly controlled by adjusting her oral drugs. After six weeks of observation, the majority of retinal hemorrhages had been absorbed, with marked improvement in her visual ability. Once the diagnosis of RAM rupture was established, direct laser photocoagulation was carefully performed at the RAM, which successfully accelerated the regression process (Figs. 2b and 3c). Three weeks after the laser photocoagulation, closure of the ruptured RAM was achieved, which was confirmed by FFA (Fig. 3b). The ERM and lamellar macular hole were noticed at her first visit and six weeks later, respectively. In addition, the lamellar macular hole was located exactly at the ILM breach in her previous visit, that is, at the junction of the VH and sub-ILM hemorrhage. We speculate that the local ILM at the edge of the lamellar macular hole was stretched and deformed due to the formation of the ERM. Coupled with the abnormal coagulation function of the patient, blood could easily pass through the deformed internal limiting membrane and thus drain into the vitreous cavity. This would be equivalent to the effect of an Nd: YAG laser membranotomy, which is also a special and interesting part of this case. Given that the patient's vision ability remained 20/40 for more than 8 months, surgery has not been considered for the time being. Furthermore, we will continue to observe this patient for a long time.

## Conclusions

Overall, we have reported a rare case of a ruptured RAM and multi-level hemorrhages caused by the Valsalva maneuver in a patient with several risk factors. We have also offered a review of relevant literature on RAMs. This patient received observation and laser photocoagulation successively and obtained satisfactory results. However, it should be noted that the patient's ILM was broken at the time of presentation, protecting her from an Nd: YAG laser membranotomy. Furthermore, if the hemorrhage is trapped under the ILM, we recommend early Nd: YAG laser membranotomy in order to drain the hemorrhage into the vitreous cavity. Once the primary lesion is exposed, further treatment should be carried out according to the patient's condition. Moreover, we stress the need for treatment guidelines for RAMs and further research to assess the long-term safety and efficacy of different treatments for RAMs.

## Data Availability

All data and materials supporting our findings are contained within this manuscript.

## Abbreviations

RAM: Retinal artery macroaneurysms
BCVA: Best-corrected visual acuity
VH: Vitreous hemorrhage
sub-ILM: Sub-inner limiting membrane
CFP: Color fundus photograph
FFA: Fundus fluorescein angiography
ICGA: Indocyanine green angiography
OCT: Optical coherence tomography
ERM: Epiretinal membrane
ILM: Inner limiting membrane
PT: Prothrombin time
APTT: Activated partial thrombin time
INR: International normalized ratio
IR: Infrared
RVO: Retinal venous occlusion
RAO: Retinal arterial occlusion
MH: Macular hole
VA: Visual ability
DR: Diabetic retinopathy
IPCV: Idiopathic polypoidal choroidal vasculopathy
CNV: Choroidal neovascularization
wAMD: Wet age-related macular degeneration
PDR: Proliferative diabetic retinopathy
anti-VEGF: Anti-vascular endothelial growth factor
tPA: Plasminogen activator
STLT: Subthreshold laser treatment
PPV: Pars plana vitrectomy
SMH: Submacular hemorrhage
